# Taurine Supplementation and Human Heat Tolerance: Mechanisms, Evidence, and Integration with Heat Acclimation, Cooling, and Hydration

**DOI:** 10.3390/nu18040592

**Published:** 2026-02-11

**Authors:** Siavash Naddafha, Jeffrey R. Stout, Cassandra Evans

**Affiliations:** 1 School of Medical & Health Sciences, Edith Cowan University, Joondalup, WA 6027, Australia; 2 School of Kinesiology & Rehabilitation Sciences, University of Central Florida, Orlando, FL 32816, USA; jeffrey.stout@ucf.edu; 3 Health Care Sciences, Nova Southeastern University, Fort Lauderdale, FL 33314, USA; ce50@nova.edu; 4 Human and Sport Performance, Rocky Mountain University of Health Professions, Provo, UT 84606, USA

**Keywords:** thermoregulation, taurine, hydration, heat, cooling, mining

## Abstract

Heat exposure during strenuous exercise increases core temperature and cardiovascular strain, impairing performance and elevating the risk of heat illness. Standard countermeasures include heat acclimation, cooling, and hydration/electrolyte planning. Taurine is a sulfur-containing amino acid present in excitable tissues and widely used as an oral supplement; emerging human trials suggest it can augment thermoregulation, primarily by enhancing eccrine sweating and evaporative heat loss. This narrative review synthesizes mechanistic and applied evidence on taurine during exercise in hot environments and evaluates potential interactions with acclimation, cooling strategies (pre- and per-cooling), and hydration practices. Across a small number of randomized, mostly double-blind crossover studies, acute (~50 mg/kg) or short-term multi-day supplementation has been associated with earlier sweat onset, higher sweat production, modestly lower core temperature (~0.3–0.4 °C), and, in one multi-arm trial, a large standardized reduction in core temperature (d ≈ 1.9), with improved exercise capacity or performance. Benefits appear to be context-dependent and may be attenuated when sweating is constrained (e.g., impermeable protective clothing) or when heat acclimation is already optimized. Because taurine may increase sweat losses, its use should be paired with individualized fluid and sodium replacement. Current evidence is promising but remains constrained by small samples and heterogeneous protocols; adequately powered field trials are required to establish dose–response, safety and efficacy across populations, and additive value when combined with established heat-mitigation strategies.

## 1. Introduction

Athletes, military personnel, and outdoor workers often face the dual challenge of intense exercise and environmental heat stress. Under such conditions, the body’s thermoregulatory capacity is pushed to its limits, raising core temperature and risking performance decrements or heat illness. To combat this, practitioners have long employed several strategies in combination, including:

**Heat acclimation**: Repeated heat exposure over 1–2 weeks triggers physiological adaptations (earlier onset of sweating, higher sweat rates, expanded plasma volume, reduced cardiovascular strain, and decreased sweat sodium content) that collectively improve heat tolerance [1,2]. These changes raise the threshold for heat intolerance, allowing acclimated individuals to perform better and more safely in hot environments. For example, a meta-analysis reported that heat acclimation can increase exercise time to exhaustion in the heat by roughly 15–20% on average [3].

**Cooling techniques**: Specialized cooling methods (pre-cooling via cold-water/ice immersion or ice slurry ingestion, cooling vests or packs, and mid-exercise cooling like cold drinks or ice towels) help lower body temperature or remove stored heat, thereby delaying fatigue. Pre-cooling before exercise often lowers starting core temperature by ~0.3–0.5 °C and can significantly improve subsequent endurance performance in the heat [4,5]. Cooling during exercise (per-cooling) can attenuate heat strain, though its efficacy may depend on environmental conditions.

**Hydration and electrolytes**: Maintaining proper hydration with water and electrolyte replacement is fundamental. Sweating is the body’s primary cooling mechanism in the heat, and adequate fluid intake sustains sweat output and cardiovascular function. Electrolyte (especially sodium) intake helps prevent imbalances and supports ongoing neuromuscular function during prolonged sweating.

In recent years, attention has turned to nutritional supplementation as a means to augment traditional heat adaptation tools. One supplement of particular interest is taurine, a sulfur-containing amino acid endogenously present in high concentrations in skeletal muscle, the heart, and the central nervous system. Taurine is well known in sports nutrition for its roles in cardiovascular function and as a common ingredient in energy drinks, but emerging evidence suggests it may also enhance thermoregulatory responses to heat stress [6,7]. Integrating taurine supplementation with acclimation, cooling, and hydration strategies may enhance heat dissipation and complement existing interventions to provide a more robust defense against heat strain.

This narrative review examines taurine’s potential role as an integrative heat adaptation strategy. We synthesize current knowledge on taurine’s mechanisms in thermoregulation, interactions with heat acclimation, potential synergistic or antagonistic effects when combined with cooling strategies, and its influence on hydration and electrolyte balance during heat stress. We also explore the translational potential of taurine for occupational and military settings, where managing heat stress is a practical concern. This review emphasizes mechanistic and applied outcomes, identifies existing knowledge gaps, and outlines directions for future research. We hypothesized that taurine primarily augments sweat-mediated evaporative heat loss and may be most effective when acclimation is incomplete and evaporative potential is preserved while offering limited benefit (or increased dehydration risk) when evaporation is constrained. By framing taurine within the broader context of heat adaptation tools, we aim to provide sports and occupational health practitioners with a comprehensive understanding of how this supplement could be leveraged to protect human performance and safety in the heat.

Literature identification and scope: To inform this narrative synthesis, we conducted targeted searches of PubMed/MEDLINE, Scopus, Web of Science, and SPORTDiscus (from inception to January 2026) using combinations of keywords including “taurine”, “heat stress”, “heat acclimation”, “thermoregulation”, “sweating”, “vasopressin”, “hydration”, “electrolytes”, and “exercise”. We prioritized human intervention studies conducted under environmental heat stress, alongside the mechanistic and applied literature on acclimation, cooling, and hydration to support integration and translation. Study identification and screening are summarized in Figure S1. Literature was managed using EndNote 21 (Clarivate, Philadelphia, PA, USA)

## 2. Taurine and Thermoregulation

Taurine is a naturally occurring sulfur-containing amino acid derivative that is widely available as an oral supplement. It is generally recognized as safe (GRAS), non-stimulatory, and cost-effective. Several physiological properties make taurine an intriguing candidate for enhancing thermoregulation. First, taurine possesses established ergogenic and fatigue-delaying effects; it has been shown to improve endurance exercise performance in temperate conditions, with performance gains (e.g., ~10% longer time to exhaustion) comparable to those from aerobic training or heat acclimation [6]. These ergogenic effects are partly attributed to taurine’s influence on calcium handling in muscle (improving excitation–contraction coupling) and its antioxidant capacity, which together can delay muscle fatigue [6,8]. Taurine supplementation also shifts substrate utilization toward greater fat oxidation over carbohydrate use [9], potentially reducing the respiratory quotient and metabolic heat production at higher intensities [10].

Next, taurine has osmoregulatory and vasoactive properties. Taurine acts as an osmolyte that helps regulate cell volume and osmotic pressure. It is released from muscle cells during exercise as an osmotic response to cell swelling, aiding fluid balance between compartments [11]. Taurine can also influence blood vessel tone; some studies in animals [4] suggest taurine has vasoactive effects (e.g., enhancing endothelium-dependent dilation in rat aorta), although the net impact on human skin blood flow during heat stress is still unclear [12]. Notably, in a controlled heat stress trial, taurine supplementation did not significantly change skin blood flow compared to the placebo, suggesting its cooling benefits arise largely from sweat evaporation rather than altered skin perfusion [7].

Taurine appears to directly affect the body’s primary heat-dissipating mechanism: eccrine sweating. Pioneering work by Page et al. [6] (2019) demonstrated that an acute dose of taurine (~50 mg/kg body mass, ingested 2 h before exercise) can enhance sweating and exercise capacity in the heat. In that randomized crossover trial, taurine increased time to exhaustion by about 10% in hot conditions (cycling at 35 °C, 40% relative humidity) and significantly elevated the end-exercise sweat rate by 12.7% compared to a placebo [6]. Participants who ingested taurine finished exercise with a slightly lower core temperature on average (38.1 °C vs. 38.5 °C in the placebo trial) and reported lower ratings of perceived exertion in the later stages of exercise [6]. These findings provided the first direct evidence that taurine can bolster thermoregulatory processes—notably by amplifying sweat evaporation and attenuating heat storage during exercise. Building on this, Peel et al. [7] conducted a controlled trial where participants took taurine daily for 8 days and then performed low-intensity exercise in a hot environment. Taurine supplementation led to an earlier onset of sweating and more prolific sweat production: whole-body sweat loss increased by ~26–27% relative to the placebo, local sweat rate by ~8–15%, and the number of active sweat glands recruited was 22–32% higher with taurine [13]). This enhanced sudomotor activity translated into a 27% greater evaporative heat loss and a 72% reduction in net heat storage during exercise, effectively improving the body’s thermal balance and reducing the rise in core temperature [7,14]. Notably, taurine extended the duration for which participants could maintain thermal equilibrium: it delayed the upward inflection in core temperature that signifies the onset of uncompensable heat gain. In other words, those who took taurine tolerated a higher level of heat stress (in this protocol, progressively increasing humidity at a fixed metabolic heat production) before their core temperature began to rise uncontrollably. Quantitatively, taurine increased the critical environmental vapor pressure (P_crit) at which heat balance could no longer be sustained from ~21.7 mmHg (placebo) to ~25.0 mmHg (taurine)—a meaningful shift indicating improved heat tolerance [7]. In practical terms, this means a taurine-supplemented individual could withstand more humid or extreme conditions before reaching a dangerous core temperature threshold.

The mechanisms by which taurine induces these thermoregulatory benefits are an active area of investigation. Several proposed pathways are plausible, but they should be interpreted as mechanistic hypotheses rather than confirmed mediators in humans. A leading hypothesis involves taurine’s action as a neuromodulator in the hypothalamus, the brain’s thermoregulatory control center. Taurine can activate glycine and GABA_A receptors, inhibitory neurotransmitter systems that may influence the threshold for heat-dissipation responses. Research in animal models supports this idea: exposing rabbits to heat stress triggers a significant release of taurine (and GABA) in the hypothalamus and cerebrospinal fluid, suggesting these act as endogenous “cryogens”—signals to counteract excessive hyperthermia [11]. Furthermore, pharmacological studies have shown that taurine acting in the brain can induce hypothermic effects. For instance, direct infusion of taurine into the brain of rodents attenuates fever and hyperthermia, indicating that central taurine can activate cooling responses and lower the body’s thermal set-point [12]. In humans, the observation of an earlier onset of sweating with taurine (i.e., sweating initiated at a lower core temperature than normal) is consistent with a downward shift of the hypothalamic set-point for initiating heat loss, analogous to what a “coolant” neurotransmitter might accomplish; however, direct neuroendocrine measurements confirming this pathway in humans are currently lacking. Taurine is known to cross the blood–brain barrier via specific transporter proteins [13], so an oral dose could feasibly reach the hypothalamus and modulate thermosensitive neural circuits.

Another proposed mechanism is hormonal modulation via arginine vasopressin (AVP), also known as antidiuretic hormone. AVP is well known for its role in water conservation at the kidney, but it may also influence sweating under some conditions: high plasma AVP levels (such as when dehydrated) have been associated with more concentrated sweat (higher sodium content) and possibly a lower sweat rate, as the body attempts to conserve fluid [14]. There is some evidence that taurine blunts AVP release or action, potentially by activating glycine receptors in osmoregulatory brain regions and creating a state sensed as “less dehydrated” [15,16]. If taurine suppresses AVP, sweat glands might reabsorb less water (because less antidiuretic signal is present), leading to more copious sweat output. In fact, classic experiments have shown that administering exogenous vasopressin can sharply reduce the sweat rate (on the order of a 50% reduction in sweat output in rats given AVP) and reduce sweat gland recruitment [17,18]. Conversely, lowering vasopressin signaling could remove this “brake” on sweating. By this reasoning, taurine’s tendency to increase sweat volume could be explained in part by an AVP-antagonizing effect—essentially allowing sweat glands to secrete more fluid onto the skin surface. It should be noted, however, that studies manipulating AVP in humans have yielded mixed results on the sweat rate [14], and no taurine–heat trial to date has directly measured AVP alongside sweating. Therefore, AVP-related statements should be considered speculative and context-dependent, and future work needs to explicitly separate what is empirically demonstrated from what is mechanistically inferred. Nonetheless, the combination of a lowered sweating threshold and an increase in sweat gland output provides a plausible explanation for taurine’s ability to provoke substantial sweating without a corresponding early rise in core temperature [6,7]. Taurine may help the body start cooling sooner and more effectively as heat stress develops, but the dominant mediators in humans remain to be confirmed.

Beyond sweating, taurine’s other physiological effects may indirectly support thermoregulation. Its antioxidant properties can protect tissues from heat-induced oxidative stress (a common issue during prolonged heat exposure), thereby preserving cellular function and possibly delaying fatigue or heat-related cellular damage. Taurine’s role in calcium handling and cardiac muscle function might improve cardiovascular efficiency, potentially mitigating the typical drift toward higher heart rates and lower stroke volume seen during heat stress. Interestingly, in an 8-day taurine study, the heart rate during exercise was similar between the taurine and placebo conditions, suggesting that taurine’s cooling effect did not significantly alter cardiovascular strain in this scenario [7]. Taurine has also been linked to improved thermal comfort—for example, one study investigating exercise in a cold environment found that participants reported feeling “comfortably warm” more quickly when they had ingested taurine, despite no difference in core temperature, hinting that taurine may alter thermal perception or tolerance [15]. This could be a central nervous system effect on how thermal signals are processed or on subjective sensation of temperature. While the influence of taurine on skin blood flow remains uncertain (as noted, no major differences were detected in the controlled trials in the heat), taurine’s cooling benefits seem to come largely from enhancing evaporative cooling via sweat. In summary, taurine supplementation augments thermoregulation primarily by amplifying the sweating response and tweaking central thermoregulatory controls to favor heat dissipation. The practical outcomes include a delayed onset of hyperthermia, lower end-exercise core temperatures, reduced perceived exertion, and improved endurance performance in hot conditions [6,7]. These effects have been demonstrated with acute dosing (a single dose 1–2 h pre-exercise) and with short-term supplementation on the order of days. The next sections discuss how taurine’s actions intersect with other heat adaptation strategies, notably the physiological adaptations from heat acclimation, various external cooling interventions, and hydration practices to assess whether taurine can synergize with, or substitute for, these established approaches. The multifaceted physiological pathways through which taurine influences heat storage and dissipation are summarized in Table 1.

## 3. Heat Acclimation Interactions

Heat acclimation (HA) is the gold-standard process for eliciting the body’s natural adaptive responses to repeated heat exposure. Through daily bouts of exercise–heat stress (typically 60–100 min in hot conditions) over about 7–14 days, a host of beneficial physiological changes occur: an earlier onset of sweating and higher sweat rates at a given core temperature, an increase in total sweat output and sweat gland sensitivity, an expansion of plasma volume, a reduction in heart rate and cardiovascular strain for a given workload, a lower resting and exercising core temperature, and reduced electrolyte loss in sweat due to increased aldosterone-mediated sodium reabsorption [1,16]. These adaptations collectively improve the ability to dissipate heat and maintain performance. For instance, time to exhaustion in the heat can increase significantly after acclimation (often on the order of 20% longer, as noted earlier), and athletes report lower perceived exertion and discomfort when they are fully heat-acclimated [3,17].

Because taurine supplementation also enhances sweating and endurance in the heat, it is natural to ask how taurine and heat acclimation might interact. Do they produce redundant effects via similar pathways, or could their benefits be additive? To date, no published study has directly combined taurine supplementation with a formal heat acclimation program, but we can draw comparisons from separate findings to make initial predictions. Interestingly, the magnitude of some sweating improvements with short-term taurine use resembles those seen with heat acclimation. In the 8-day taurine study by Peel et al. [7], participants’ whole-body sweat loss during a standardized exercise increased by ~26% relative to the placebo condition. This is on par with the ~20–25% increase in sweat volume that is often observed after a week or two of heat acclimation [1,18]. Taurine also boosted the number of active sweat glands by about 22–32% (depending on measurement site), which is similar in magnitude to the ~28% increase in sweat gland recruitment reported after 8 days of exercise–heat acclimation in a separate study [18]. These parallels suggest that taurine can mimic some acclimation-like effects in a very short timeframe. In essence, taurine pharmacologically induces some of the same sweat-enhancing adaptations that normally require repeated heat exposures to develop.

However, not all responses overlap completely. For example, taurine increased local sweat rate by ~8–15% above placebo in Peel et al. [7] was roughly half the improvement typically seen with full heat acclimation (~20–30% increase in peak local sweat rate capacity) [1,18,19]. Moreover, heat acclimation tends to sustain a high sweating rate throughout prolonged exercise, whereas taurine’s effect in the 8-day trial was most pronounced in the early phase of exercise and then tapered as environmental strain became more extreme. Specifically, with taurine, sweating began sooner and was heavier in the initial period, but once humidity climbed to very high levels (reducing evaporative efficiency), the difference in the sweat rate between the taurine and placebo conditions narrowed [7]. In other words, taurine effectively “jump-started” the sweating response—acting like a fast-forwarded acclimation in the first minutes of heat exposure—but it did not continually elevate sweat output under the most severe conditions the way a fully acclimated system might. This temporal pattern hints that taurine and acclimation might operate via slightly distinct mechanisms or face different limits. Acclimation brings about structural and long-term physiological changes (such as sweat gland hypertrophy and increased sensitivity to internal temperature) that allow sustained high sweat rates even as core temperature rises. Taurine’s benefit appears to involve lowering the threshold to start sweating (a central set-point change) and possibly removing some acute hormonal inhibition (e.g., AVP effects), without necessarily increasing the maximal capacity of sweat glands in the short term. Thus, an acclimated person and a taurine-supplemented but unacclimated person might both start sweating quickly when exercising in the heat, but the acclimated individual may achieve a greater absolute sweat output under prolonged heat stress and maintain thermal stability longer once fully taxed.

These considerations suggest potential complementarity between taurine and acclimation. Taurine could be particularly useful in scenarios where an athlete has limited time to acclimatize. For example, if someone travels to a hot competition and has only a few days to adapt, taking taurine might confer some immediate thermoregulatory aid akin to partial acclimation. It essentially “fills the gap” by triggering earlier and more robust sweating in those first critical sessions or events before true physiological acclimation has had time to develop. Over the course of an acclimation program, taurine might also enable athletes to train harder or more comfortably in the heat by keeping them slightly cooler or lowering their perceived exertion. This could theoretically accelerate the acclimation process itself: if taurine supplementation reduces cardiovascular strain or thermal discomfort, an athlete might tolerate a higher training intensity or volume in each heat session, potentially stimulating greater adaptation (though this remains speculative). An analogy can be drawn to how some athletes use pre-cooling during heat training—by lowering initial strain, they can achieve better quality workouts and thus adapt more. Taurine might similarly act as a tool to enhance training capacity in hot conditions.

On the other hand, if an athlete is already fully heat-acclimated, the marginal gains from taurine may be smaller. A well-acclimated individual already has a low internal threshold for sweating and maximized sweat output; taurine might still nudge the sweating threshold a bit lower or improve comfort, but the “room for improvement” is less than in an unacclimated state. It is likely that taurine’s largest impact would be seen in non-acclimated or only partially acclimated individuals who need an extra boost in heat tolerance. For instance, a highly acclimated athlete could still consider taurine before an important competition in extreme heat, but they should temper expectations: it might help reduce peak core temperature or perceived exertion slightly, but it probably will not confer another 10% endurance boost on top of their acclimation [6]. Indeed, many of taurine’s benefits (earlier sweating, expanded plasma volume, improved exercise capacity in heat) overlap with what acclimation already provides. Once those adaptations are in place, taurine might function as a fine-tuning aid or an “insurance policy” on race day, rather than a game-changer.

It is also important to note that acclimation brings about adaptations beyond what taurine addresses. For example, heat acclimation induces sweat gland conservation of sodium (via aldosterone) such that sweat becomes more dilute in an acclimated person. Taurine’s mechanism does not inherently include sodium retention; if anything, by increasing sweat volume without altering sweat sodium content, taurine could lead to greater absolute salt losses if hydration is not adjusted (more on this in the hydration section). Similarly, acclimation improves cardiovascular stability (e.g., maintaining blood pressure and central blood volume) and increases heat shock protein expression for cellular protection—areas where taurine’s contributions, if any, are not fully understood. Therefore, taurine is not a replacement for comprehensive heat acclimation but rather a complementary aid. At best, we can view taurine as a tool to augment heat acclimation or to provide acclimation-like benefits when full acclimatization is not feasible.

In summary, taurine and heat acclimation both independently enhance an individual’s tolerance to heat stress, primarily by improving the sweating response and reducing thermal strain. Short-term taurine supplementation can achieve on the order of 50–80% of the sweating improvement that a traditional acclimation program would yield, at least in the initial phase of exercise [6,7,18]. Combining them could be beneficial: use taurine as a stopgap or booster in the early days of heat exposure, and continue traditional acclimation protocols for sustained, long-term adaptation. Future studies should directly explore this interaction. For example, researchers could examine whether taking taurine during a heat acclimation program leads to greater final adaptations (does it allow one to achieve a higher acclimated sweat rate or larger plasma volume increase?) or whether taurine could help maintain acclimation adaptations during periods away from heat (perhaps by simulating the sweating response intermittently). Another question is whether taurine can act as a “heat acclimator in a pill” for individuals who cannot adequately acclimatize due to geographic or scheduling constraints. Clarifying these points will inform coaches and practitioners on how best to integrate taurine into training plans. At present, an evidence-based approach might be as follows: if facing an imminent heat challenge with suboptimal acclimation, consider taurine supplementation (e.g., ~3–6 g daily, or 50 mg/kg) to gain some of the sweating and comfort benefits while understanding it is one piece of the puzzle. And for those who are well acclimated, taurine can still be a modest additional layer of protection on competition day, but core strategies like proper heat training, cooling, and hydration remain paramount.

## 4. Cooling Strategies: Interactions with Taurine (Ice Slurries, Cold Water Immersion, Cooling Garments)

While acclimation and taurine primarily act on the body’s internal heat-dissipation mechanisms, cooling strategies provide external means of reducing heat strain. Athletes frequently combine multiple approaches, for example, pre-cooling the body before an event, using cooling aids during exercise, and taking taurine or other supplements—so it is important to understand how these might interact. Key cooling methods include pre-exercise cooling (via cold-water or ice immersion, ice slurry ingestion, cooling vests, etc.), per-exercise cooling (such as drinking cold fluids, applying ice packs or wet towels during breaks, wearing cooling garments during activity), and post-exercise cooling for recovery. The goal of any cooling intervention is to lower body temperature (or increase the capacity to store heat) so that the athlete can perform longer or at higher intensity before overheating.

Pre-cooling involves reducing core and skin temperature before exercise begins. Techniques like cold-water immersion (sitting in 15–20 °C water for 20–30 min) or wearing an ice-cooled vest before exercise can decrease baseline core temperature by a few tenths of a degree and significantly improve subsequent endurance performance in the heat [4,5]. For example, cold-water immersion is one of the most effective pre-cooling methods due to water’s high thermal conductivity; athletes commonly see performance improvements (longer time to exhaustion or faster time-trial times) after immersing in cool water. Ice slurry ingestion—drinking a semi-frozen beverage (~−1 to 0 °C, often flavored ice slush), has also become popular because it can both lower core temperature (by cooling blood in the stomach and absorbing heat as the ice melts) and increase the body’s heat storage capacity (the ingested ice must absorb heat to warm to body temperature) [20]. Some studies have found that a well-timed ice slurry pre-cool can extend exercise endurance nearly as much as water immersion, with the added practicality that it does not require special equipment [4,20]. A meta-analytic review concluded that pre-cooling, on average, has a meaningful positive effect on endurance performance in hot conditions—particularly when using cold-water immersion or combining multiple cooling methods [4].

One known side effect of pre-cooling, however, is that it can delay the activation of the body’s own heat-loss responses once exercise starts. If an athlete begins exercise with a cooler core and skin, the hypothalamus senses less urgency for heat dissipation, so the onset of sweating and skin vasodilation can be blunted or delayed in the initial phase of exercise [17]. Essentially, pre-cooling “tricks” the body into thinking it is not hot yet, so it holds off on sweating. This means that although the athlete starts at a lower core temperature, their core temperature might rise faster than normal once they begin exercising, until their body eventually realizes heat is accumulating and then ramps up sweating. In practice, this is usually not problematic—the cooling benefit still outweighs the cost, because by the time core temperature catches up, the athlete has banked extra minutes of safety/performance. But it does illustrate an important point: pre-cooling reduces initial heat strain at the expense of a slower reflexive thermoregulatory response.

Now, consider taurine’s effect in this context. Taurine, as discussed, lowers the internal threshold for sweating—it makes you start sweating at a lower core temperature than you normally would. This is effectively the opposite of what pre-cooling does to the reflex threshold. Therefore, combining pre-cooling and taurine could yield an interesting synergy: The pre-cooling will still provide a buffer by reducing the starting core temperature, but the presence of taurine might counteract the delay in sweat onset by ensuring that as soon as the core temperature begins to rise even slightly, the sweat glands activate. In theory, an athlete who both pre-cools and takes taurine would begin exercise with a cold core and still start sweating relatively early in the exercise bout (because taurine pushes the hypothalamus to trigger sweating sooner than it otherwise would in a cool body). There is not yet direct research on this exact combination, but from a physiological standpoint, it could be advantageous. The athlete essentially gains a dual advantage: a greater heat storage capacity via a lower starting temperature and a prompt, vigorous sweat response to manage metabolic heat. This combination could extend the time to reach critical core temperatures more effectively than either strategy in isolation. A key nuance is the interaction between these states; while taurine lowers the threshold for sweating, it does not bypass the body’s requirement for an initial rise in internal heat. If pre-cooling has significantly depressed the core temperature, the sweat glands will remain inactive until exercise-induced heat production reaches the “taurine-adjusted” baseline. Once that threshold is met, however, the lowered set-point ensures that evaporative cooling and skin blood flow ramp up aggressively. The result is a slower initial rise in core temperature followed by a highly responsive cooling mechanism once the heat load accumulates.

Cooling during exercise (per-cooling) includes strategies like ingesting cold fluids or ice slurries during activity, applying ice/cold towels during breaks (e.g., halftime of a game), fanning oneself, or wearing cooling garments while moving. These strategies aim to remove heat as it is being produced, rather than solely beforehand. Internal cooling via cold drinks is common and can lower core temperature modestly if done continuously. However, a phenomenon often observed is that when an athlete drinks a cold fluid and it cools the core even slightly, the body may reflexively reduce sweat production because the perceived need for sweating is reduced [21]. In conditions where sweat evaporation works well (a hot–dry environment with good airflow), this compensation can negate much of the benefit of the cold drink: the athlete might feel cooler from the drink, but because they then sweat a bit less, the total cooling (drink + sweat) may end up similar to not having the drink at all (if the sweat reduction offsets the drink’s cooling) [4,21]. In contrast, in hot–humid conditions or whenever sweat evaporation is impaired (for instance, no wind or wearing heavy clothing), sweat is not very effective anyway, so a cold drink provides a net cooling that sweating could not achieve. In those cases, internal cooling during exercise is more beneficial.

How might taurine factor in here? If taurine has primed the body to sweat more, and the athlete also consumes an ice slurry or cold drink during exercise, there could be a push–pull effect. In a hot–dry scenario, taurine on its own would cause abundant sweating, maximizing evaporative cooling. If the athlete adds a cold drink, the body might sense the cooling and try to throttle back the sweat rate to avoid dropping the core temperature too much (the body tends to defend against over-cooling). Thus, one could imagine that the extra sweat taurine would have induced might be partially reduced by the body if sufficient internal cooling is provided through drinks. This does not mean the combination is harmful; it likely means the athlete could achieve the same cooling with slightly less sweat loss, which might actually preserve fluid. But it suggests that in an optimal setting (dry heat), taurine and internal cooling might have some redundancy in their effects. On the other hand, in a hot–humid environment, taurine’s extra sweat might not evaporate well (so its cooling effectiveness is blunted), and here, drinking an ice slurry during exercise can add a cooling boost that sweat alone cannot provide. In this scenario, the combination is complementary: taurine pushes your sweating to the max (which still evaporates some heat, albeit less efficiently in humidity), and the ice slurry directly cools you from the inside, covering for sweat’s limitations. Many team sport athletes in outdoor summer events (e.g., soccer, rugby) already consume cold drinks or slushies at halftime; taking taurine before the match could ensure they are sweating efficiently in the first half, and then the mid-game cooling keeps them from overheating in the second half as conditions worsen or as they become dehydrated.

It is worth noting a practical point: If an athlete uses both taurine and aggressive cooling, they must monitor hydration status carefully. Taurine will cause them to lose more fluid through sweating, and effective cooling (especially internal cooling) can reduce thirst or the subjective need to drink. An athlete might feel relatively comfortable and cool (thanks to the cooling strategies) and not realize how much fluid they have lost through the taurine-boosted sweating. Therefore, a combination approach demands a well-planned hydration strategy: scheduled drinking of fluids (potentially with electrolytes) rather than relying purely on thirst, because thirst can be an unreliable indicator when the body is artificially cooled.

Cooling garments and external cooling devices can also interact with taurine. Athletes and workers sometimes wear cooling vests (with ice gel packs or phase-change material), cooling collars or headbands, or even chilled water circulation suits during exercise or recovery. These methods cool the skin and blood flowing through the skin, thereby extracting heat from the body. For instance, a cooling vest worn during exercise can significantly lower skin temperature and improve thermal comfort and exercise tolerance [22]. If an athlete has taken taurine and is also wearing a cooling garment, one effect might be that the skin cooling provided by the garment will reduce the drive for sweating (similar to pre-cooling). Taurine will still have the sweat glands on standby, ready to secrete, but if the cooling vest keeps the skin temperature low, the body might not need to activate as many sweat glands to achieve heat balance. In this case, taurine’s influence might be muted by the external cooling—again, not necessarily a bad outcome, as long as cooling is happening by one route or another. One interesting hypothetical is that if taurine makes an athlete sweat more without needing as much skin blood flow (because evaporative cooling is doing the job), adding a cooling vest could offload some of the heat through direct conduction, possibly allowing more blood to remain in central circulation or go to muscles. This could theoretically improve performance further by reducing cardiovascular strain. However, this is quite speculative and likely a small effect, if any.

Where combination strategies might be counterproductive is in scenarios of extreme clothing or equipment. If a person is wearing impermeable protective gear (e.g., a firefighter in a heat-resistant suit or a soldier in full chemical/biological protective attire), sweat evaporation is severely limited because sweat cannot easily escape the clothing. In such cases, making the person sweat more (with taurine) will just accumulate sweat inside the clothing, leading to faster dehydration without much cooling benefit. Someone working in those conditions is better off focusing on external cooling (like circulating cold water under the suit or cooling packs) and staying hydrated; taurine’s effect could simply be to increase fluid loss. Thus, for, one might avoid using taurine or use it cautiously. For example, an American football player wearing heavy padding in a hot–humid environment might not gain much from extra sweating (their equipment traps heat and sweat), so their team might rely more on external cooling like misting fans and ice towels during timeouts. In contrast, a soccer player with minimal clothing in the same heat could benefit greatly from extra sweating because their sweat can evaporate freely.

In summary, taurine can be combined with cooling strategies, and doing so can yield additive benefits in many circumstances, but the interactions are nuanced. An integrative approach for, say, an endurance athlete might be to take a dose of taurine ~1–2 h pre-race to enhance internal cooling capacity, implement a pre-cooling routine (e.g., ice slurry or cold tub) before the start, and then have cold drinks or ice available during the event if possible. The taurine ensures that once the pre-cooling wears off, the body’s sweating kicks in at full force to continue cooling, and the intermittent cooling helps remove heat during critical moments (like transitions or rest stops), especially if environmental conditions limit sweat effectiveness. Coaches and sport scientists should be mindful of the balance: If the athlete is overdoing cooling to the point where they barely sweat (which is unlikely except in short events), taurine’s advantage might not manifest fully. But in most real settings, heat production will eventually overwhelm external cooling, and this is when taurine’s enhanced sweating can make a difference. Since no studies have explicitly tested “taurine + cooling” vs. either alone, this is an area ripe for research. Such studies could measure the core temperature, sweat rate, performance, and hydration status in groups using both interventions versus single interventions. They would help confirm whether taurine provides a noticeable edge when an athlete is already employing state-of-the-art cooling strategies, or if the benefits are simply redundant. Based on current understanding, we expect taurine to be most beneficial when internal heat dissipation needs to be bolstered (i.e., when external cooling cannot fully handle the heat load), which is often the case in longer-duration exercise or competitions where continuous cooling is not feasible. In those scenarios, combining taurine’s internal cooling boost with external cooling methods likely offers maximal protection against overheating.

## 5. Hydration and Electrolyte Considerations

Hydration is the foundation that underpins all other heat adaptation strategies because sweating, the body’s main cooling engine, depends on fluid availability. Any intervention that increases sweating (whether heat acclimation or taurine supplementation) will also increase fluid requirements and the risk of dehydration if fluid intake is not adjusted accordingly. Thus, understanding how taurine influences fluid and electrolyte balance is essential for its safe and effective use as a heat adaptation tool.

**Fluid loss and plasma volume**: By causing a person to sweat more, taurine will increase total fluid loss for a given exercise bout if drinking is held constant. Indeed, in the 8-day supplementation study, participants in the taurine trial lost more body mass through sweat than in the placebo trial when exercising under identical conditions [7]. One might assume that losing more sweat would lead to a greater contraction of plasma volume (since sweat is drawn from plasma). Interestingly, however, Peel et al. [7] found that by the end of exercise, the taurine and placebo conditions showed no significant difference in plasma volume reduction, despite the extra ~0.3 kg of sweat loss with taurine. Both trials ended with a mild plasma volume contraction (as expected from any dehydrating exercise in heat), but taurine’s trial did not result in a worse plasma volume status than the placebo. This suggests that taurine might have some capacity to help maintain the plasma volume even as it increases sweat output. One hypothesis is that taurine, being an osmolyte, could pull fluid into the vascular compartment to offset sweat losses. When taurine is ingested and absorbed, the plasma taurine concentration rises, which would increase plasma osmolarity slightly and draw water into the bloodstream from the intracellular and interstitial spaces [4]. Essentially, taurine could be acting like a mild plasma expander initially. In theory, this provides a larger reservoir of fluid in the circulation that sweating can tap into, thereby preserving blood volume and delaying cardiovascular drift (an excessive heart rate increases due to falling stroke volume). Some researchers have speculated that taurine release from muscles during exercise is a natural mechanism to aid fluid shifts—muscle cells expel taurine as they swell, which then helps retain water in blood and interstitial fluid [4].

However, it is important to keep this effect in perspective. The primary drivers of fluid shifts and plasma volume maintenance during exercise are the major osmolytes like sodium and proteins; taurine, while osmotic, is a small player relative to these. For example, the plasma sodium concentration and total electrolyte content largely dictate how water distributes between compartments. Classic studies have shown that sodium losses and gains have a direct impact on the plasma volume [4,19]; those which studied taurine’s role in osmoregulation during exercise concluded that although taurine levels change with endurance exercise and heat, taurine alone does not govern plasma volume changes—those are primarily controlled by sodium and fluid balance [7]. In this trial, the fact that the plasma volume ended up being similar with more sweating could be partly due to their measurement timing and individual variability. It is possible that taurine caused a slightly higher plasma volume pre-exercise (via osmotic water retention) or triggered some acute renal water retention that evened out losses. But over the course of an hour of exercise, any advantage could have been subtle. The takeaway for athletes is that taurine should not be assumed to protect against dehydration. Even if taurine temporarily buffers plasma volume, any extra sweating it induces must ultimately be compensated by fluid intake. Athletes using taurine in the heat should plan to drink proportionally more to match their increased sweat losses. A rough approach could be to monitor body mass changes: if you normally lose 1.0 kg in a training session but with taurine you lose 1.3 kg, then you know you need to drink that much more to stay equally hydrated. Thirst can serve as a guide, but thirst might not always keep up with an artificially elevated sweat rate, so conscious drinking schedules or monitoring are wise.

Electrolyte balance: With increased sweating comes increased loss of electrolytes, especially sodium and chloride. A well-acclimated person’s sweat is more diluted (they lose less sodium for a given volume of sweat) because acclimation upregulates sweat gland sodium reabsorption under the influence of aldosterone. In an unacclimated person, sweat can be quite salty, and heavy sweating over hours can lead to substantial sodium depletion if not replaced. To date, there is no direct published data on whether taurine changes the sweat electrolyte concentration; mechanistic links via AVP have been proposed but remain unconfirmed in taurine–heat trials, and AVP–sweat relations in humans are mixed [14]. Regardless of concentration, total sodium loss will generally rise when the sweat volume rises. For athletes, this means that when using taurine, electrolyte replacement needs to be planned proactively, particularly during prolonged exercise, repeated sessions, or multi-day heat exposure. In practice, this can involve using sodium-containing sports drinks during exercise, consuming salty foods or electrolyte supplements after exercise, and monitoring body mass change and symptoms to avoid both under-replacement (cramps, fatigue) and over-dilution. Neglecting sodium replacement can increase risk of exercise-associated hyponatremia if large volumes of plain water are consumed after heavy sweating. This becomes more pertinent if taurine is used repeatedly across training camps, tournaments, deployments, or work blocks, where cumulative sodium deficits can develop.

Taurine itself is sometimes included in rehydration drinks or formulations, under the idea that it might aid “cellular hydration.” Taurine can modulate the movement of ions like potassium and calcium across cell membranes and has membrane-stabilizing effects [23]. By helping cells regulate their volume, taurine might prevent cells from shrinking too much when extracellular fluid is lost or from swelling during rehydration. There is some speculation that taurine could reduce muscle cramps or heat cramps by stabilizing excitable membranes and balancing calcium—though this is not conclusively proven. Anecdotally, taurine has been used in some clinical settings to manage electrolyte disorders, but athletes should primarily focus on the basics: replacing sodium, chloride, and water lost in sweat.

Another subtle aspect is the potential effect of taurine on thirst and drinking behavior. If taurine does suppress AVP or alters central thermal perception, one might wonder whether taurine could reduce perceived thirst or discomfort relative to physiological strain. In the controlled trials, fluid intake was fixed or controlled, so we do not have data on spontaneous drinking. It would be wise for athletes and heat-exposed workers not to rely solely on thirst or subjective comfort particularly if taurine reduces RPE in late exercise [6] because lower perceived strain could delay self-regulation (e.g., slowing down, seeking shade, or drinking). A sensible approach is to drink at regular intervals and observe urine color/volume and body weight changes to gauge hydration status, rather than assuming, “I’m not thirsty, so I must be fine.”

Fortunately, taurine is unlike caffeine in that it is not a diuretic and does not increase urine output during exercise. During exercise in the heat, the body naturally suppresses urine production via high AVP levels; taurine might lower AVP a bit, but probably not enough to produce clinically meaningful diuresis during heavy exercise (the exercise stress itself dominates in that scenario). If anything, taurine’s primary fluid-loss pathway is via sweat, not urine. Some studies in resting conditions or cold have noted minor differences in fluid distribution with taurine but nothing that would substitute for standard hydration and electrolyte practices [19].

In practical terms, athletes using taurine in hot conditions should take the following steps to ensure proper hydration and electrolyte balance:

**Begin well hydrated**: Before exercising in the heat (with or without taurine), ensure you are rehydrated. This may involve drinking sufficient fluids the day and morning before and including some salt in pre-event meals to boost plasma volume. Taurine supplementation does not eliminate the need for a proper hydration status at the outset.

**Adjust fluid intake during exercise**: Recognize that you may be sweating more than usual with taurine. Plan to drink a bit more frequently or in greater volume. Using a cold or cool drink can also serve dual purposes of cooling and hydrating but remember not to let the cooling effect fool you into under-drinking.

**Include electrolytes**: Especially for longer-duration exercise (>60–90 min) or repeat sessions, use sports drinks or add electrolyte tablets that provide sodium (and some potassium) to your fluids. A common guideline is to ingest around 300–600 mg of sodium per hour during heavy sweating, though needs vary widely. Taurine’s impact on sweat output means your sodium loss per hour could be correspondingly higher, so electrolyte replacement is important to prevent cramps or hyponatremia.

**Monitor recovery and rehydration**: After exercise, replace 100–150% of the fluid lost (for example, if you lost 1 kg body mass, drink about 1 to 1.5 L of fluids over the next few hours). Include salty snacks or oral rehydration solutions to expedite the restoration of plasma volume. Watch for any signs of dehydration (dark urine, elevated resting heart rate) or overhydration (very clear urine, bloating); both are to be avoided. Taurine might slightly help retain fluid by drawing water into cells and blood, but normal rehydration protocols should still be followed.

In summary, taurine can be thought of as a tool that increases evaporation for cooling. It makes the sweating “engine” run faster, which is great for cooling as long as the fuel (water and electrolytes) is supplied. It might have a minor effect in conserving plasma volume via osmotic action, but athletes should not rely on this, it is far more important to consciously manage hydration. Some evidence even suggests that taurine could mitigate the subjective discomfort of heat (making one feel better despite rising body heat), which could be a double-edged sword: you feel fine but could be quietly losing a lot of fluid. Therefore, objective measures (weigh-ins, planned drinking) are valuable when integrating taurine into a heat stress scenario. With a sound hydration and electrolyte plan, taurine’s benefits on cooling can be safely realized without tipping into dehydration or electrolyte imbalance. Example fluid and sodium adjustment strategies when using taurine in the heat are provided in Table S4.

## 6. Translational Potential in Occupational and Military Settings

Most research on taurine and thermoregulation to date has focused on athletic performance in controlled experiments. Translating these findings to occupational and military settings is conceptually appealing, but it should be made explicit that most empirical evidence currently comes from laboratory-based protocols in relatively small samples of young, healthy adults. Field effectiveness (e.g., in real work cycles, variable pacing, protective equipment, and operational constraints) requires confirmation in ecologically valid trials. Nonetheless, the underlying physiological targets—sweating, evaporative heat loss, and thermal balance—are relevant across sport, military, and physically demanding work.

A major attractive feature of taurine for broad use is its accessibility and safety profile. Taurine is inexpensive and readily available over the counter as a dietary supplement. It naturally occurs in foods (especially meat and seafood) and in popular energy drinks, and it is not considered a drug or controlled substance. Research and regulatory reviews have generally found taurine supplementation to be safe at commonly used doses; even chronic intakes of 3–6 g per day for several weeks have shown no serious adverse effects in healthy adults [24]. Doses as high as 10 g/day have been tested in short trials without ill effects, though typically 1–3 g/day is sufficient for ergogenic purposes [24]. Furthermore, taurine is not banned by sports organizations or military policies—it does not appear on WADA’s prohibited list, for example. For the military, this means it could be given to service members without legal or ethical concerns (unlike certain pharmaceuticals or stimulants that raise questions). For occupational use, it could be provided as a simple nutritional aid, akin to how some jobs provide electrolyte packets or cooling vests for heat safety.

The potential benefits of taurine in these settings mirror those observed in athletes (e.g., greater sweating and evaporative heat loss, improved thermal balance, and delayed onset of uncompensable heat strain); however, these proposed applications should be framed as promising but preliminary until confirmed in field-based studies. In particular, any strategy that increases sweat output must be paired with operational hydration and electrolyte planning to avoid unintended dehydration or sodium depletion during prolonged shifts or multi-day exposures.

However, occupational and military uses come with unique challenges and considerations:

**Clothing and equipment**: As mentioned earlier, many military and occupational scenarios involve wearing heavy or protective clothing that severely limits evaporative cooling. A soldier in body armor and uniform, or a hazmat worker in an encapsulated suit, is in a very different situation from a shirtless runner. In these cases, the efficacy of taurine’s main benefit (increasing sweat) could be significantly reduced. If sweat cannot evaporate due to protective gear, the extra sweat from taurine will mostly be wasted (it will just soak the clothing and drip away, providing minimal cooling). This could potentially worsen dehydration risk without much thermal benefit. Therefore, the use of taurine in such scenarios would need to be paired with strategies to facilitate evaporation when possible (like ventilating the suit periodically or using wicking underlayers) or to provide alternative cooling (e.g., microclimate cooling units). For instance, modern militaries sometimes employ microclimate cooling systems for troops in extreme gear (vests with cooling packs or chilled water circulating through a vest under armor). Taurine could complement these—keeping the soldier’s internal threshold low so they start to sweat as soon as they get any chance for evaporation (like when they temporarily remove a helmet or open a jacket), while the cooling unit handles heat during enclosed periods. Occupational health practitioners would need to assess on a case-by-case basis whether the workforce can actually benefit from more sweating. In many typical jobs (construction, agriculture, etc.), workers wear breathable clothing and can sweat freely—here, taurine might shine. In other cases (firefighters in turnout gear during an active fire), sweat evaporation is near zero and the focus must be on external cooling and hydration; taurine’s role might be minimal or relegated to post-incident recovery when gear is off.

**Logistics and compliance**: Implementing a supplementation protocol in the field requires practicality. One advantage of taurine is its simplicity: it could be as easy as giving people a few tablets or a taurine drink with their morning meal. The timing is important—most studies suggest taking taurine about 1–2 h before the heat exposure for peak effectiveness [6]. In a military setting, this could be incorporated into the daily routine (e.g., command could issue a standard 2–3 g taurine dose with breakfast on days of expected heat stress). Because taurine is odorless and fairly tasteless (if in capsule form), compliance might be reasonably good, especially if soldiers are educated on its purpose. Unlike some strategies that require infrastructure (like setting up cooling stations or scheduling extra breaks), taurine is a lightweight, individual-level intervention. This makes it attractive for remote or undeveloped operational areas. However, ensuring consistent use might require leadership emphasis and perhaps monitoring (just as troops are often required to take salt tablets or measured water intake in some situations).

**Individual variability and monitoring**: Not everyone respond identically to heat or to supplements. In a platoon or a work crew, some individuals may naturally tolerate heat well (strong sweat responders, high fitness, etc.) and some poorly. It is possible that taurine could benefit the latter group more, for example, those who do not sweat much might get a needed boost. But there could also be “non-responders” to taurine who see little change. In an operational trial, it would be wise to monitor a few metrics initially: perhaps measuring soldiers’ core temperatures or heart rates during training with and without taurine or simply tracking reports of heat exhaustion symptoms. If someone taking taurine still overheats, it is not a magic shield, they would need other interventions. Conversely, if it appears to help the majority (fewer complaints of overheating, better endurance on marches), it could be formally adopted. Any large-scale use should come with education: personnel must understand that taurine is not a license to overexert or ignore heat safety protocols. It is an aid, not an invincibility potion. Commanders and safety officers would still enforce work–rest cycles, water intake, and buddy systems for spotting heat illness. One could imagine a scenario where, because everyone took taurine, a unit might push a bit harder in training, this could be beneficial if done smartly (enhancing acclimation and fitness), but it could also lead to risk if overdone. Thus, integrating taurine would best be done alongside existing heat stress management guidelines [2,25], not as a replacement for them.

Combination with other aids: Military and occupational contexts sometimes involve other ergogenic aids or stimulants. For example, personnel on long missions might use caffeine or modafinil to stay alert. Caffeine can increase heat production and, in some individuals, can modestly alter fluid balance; combining multiple agents that shift perception (lower RPE) or thermoregulatory responses could complicate self-pacing and heat-symptom awareness. Athletes and soldiers may also use beta-alanine; because beta-alanine and taurine share transporter pathways, there is a theoretical basis for interaction (e.g., altered tissue taurine availability), but practical implications in the heat are not established. Accordingly, co-ingestion should be approached conservatively in high-risk heat settings, with emphasis on hydration, sodium replacement, and monitoring for atypical fatigue, cramping, or blunted warning signs.

**Ethical and long-term considerations**: Providing taurine to large groups as a preventive measure raises the question of long-term use. If a soldier is in a desert for 6 months, would they take taurine every day? We do not yet know if chronic daily use over months retains the benefits or if tolerance develops. It will be important to study whether daily taurine for long periods has any unforeseen effects (positive or negative). On the ethical side, taurine is naturally present in the diet and not a drug, so it is more akin to giving vitamin D to people in low-sunlight postings—it is generally acceptable. Nonetheless, obtaining informed consent in some occupational contexts might be considered (especially if done as a study first). Once proven, it could just become a recommended nutritional practice.

From a broader perspective, as climate change leads to more frequent heat waves and higher global temperatures, interventions that can help humans cope with heat will be increasingly valuable in many sectors. Taurine, if validated in field trials, could become part of the standard toolkit for heat management along with acclimation programs, cooling devices, hydration protocols, and educational initiatives. For example, a mining company operating in tropical conditions might implement a heat stress prevention program that includes: heat acclimatization for new workers, scheduled hydration breaks with electrolyte–taurine beverages, cooled rest areas, and monitoring of worker core temperatures. Similarly, an army might include taurine supplements in their field rations for deployments in hot regions, accompanied by instructions on usage and continued emphasis on traditional countermeasures.

In summary, the translational potential of taurine to occupational and military settings is promising due to its practicality and the alignment of its effects with the needs of these populations. It essentially offers a nutritional, user-friendly method to crank up the body’s cooling system. However, effective translation will require real-world studies to confirm benefits outside the lab and to establish protocols (appropriate dosing schedules, any contraindications, etc.). If these studies are successful, taurine could be integrated into heat stress management policies, helping protect those who work in the heat not for sport but as a necessity. By reducing heat-related risks and improving endurance, taurine supplementation might ultimately enhance both the safety and productivity of workers and the operational effectiveness of military personnel in hot environments.

## 7. Limitations and Future Directions

While the integration of taurine into heat adaptation strategies is compelling, current knowledge has limitations. Key conclusions are presently informed by a small number of human intervention studies (approximately 2–3 primary trials under environmental heat stress), typically with modest sample sizes and predominantly young, healthy participants in controlled laboratory conditions. As such, interpretation should clearly distinguish demonstrated outcomes (e.g., increased sweating, improved heat-balance metrics, and performance changes in specific protocols) from mechanistic hypotheses (e.g., AVP modulation, hypothalamic set-point shifts) that require direct confirmation. Key limitations and future research directions are described below:

Most studies have used a dose of around 50 mg/kg of body mass (approximately 3–4 g for a 75 kg person) taken 1.5–2 h before exercise, based on the time needed for taurine to peak in plasma [10]. It is unclear whether lower doses might also be effective or if higher doses could yield greater benefits (with diminishing returns or potential side effects). There may be a ceiling effect where excess taurine is simply excreted without added benefit. Research should map out the dose–response curve for taurine’s thermoregulatory effects. Additionally, the duration of effect is not fully documented—taurine levels return to baseline roughly 6–8 h after ingestion [24]. In a scenario of all-day work in heat, we need to know if a single morning dose suffices or if multiple smaller doses spaced out (e.g., morning and midday) are better to sustain the effect. Chronically, would taking taurine everyday lead to any tolerance or sustained changes in baseline thermoregulation? These questions remain open.

Related to the above, all current evidence comes from acute (one-off) or short-term (up to 1–2 weeks) supplementation. If athletes or workers take taurine daily over a season or a deployment, do the benefits persist? There is a possibility of physiological adaptation—for example, the body might adjust taurine transporter levels or receptor sensitivity after chronic high intake, potentially reducing effectiveness. Conversely, some adaptations might be enhanced (one could speculate that consistently lower heat strain could result in a more sustained training stimulus or better recovery). It is also vital to confirm safety over longer periods; while short trials show no issues, long-term high-dose taurine has not been extensively studied in heat-stressed individuals. Future research could involve multi-week field studies where participants use taurine throughout a summer training camp or work season, monitoring not just performance and thermoregulation but also general health markers.

The exact mechanisms by which taurine exerts its effects need clarification. While central neurotransmission and AVP modulation are hypothesized, direct evidence in humans is limited. Future studies could measure changes in plasma AVP levels or osmoregulatory hormones with taurine supplementation during heat exposure. If taurine truly blunts AVP, we should detect a difference in either AVP concentration or downstream effects (like urine concentration or sweat sodium content). Neuroendocrine studies, perhaps involving cerebrospinal fluid sampling or advanced imaging, could explore whether taurine is altering hypothalamic activation patterns during thermal stress. Additionally, examining sweat composition (electrolyte and protein content) in taurine vs. placebo trials would be insightful to see if gland function is altered beyond just volume. Animal models will continue to be useful for invasive mechanistic experiments: e.g., using pharmacological blockers to see if glycine or GABA receptor antagonists negate taurine’s cooling effect or genetically modifying taurine transport to see how this affects thermoregulation. Understanding mechanisms is not just an academic exercise, it could help identify whether taurine can be synergistic with other interventions (for example, if taurine works mainly via central pathways, combining it with a peripheral enhancer might be especially potent).

Athletes and workers often consume other supplements (caffeine, electrolytes, adaptogens, etc.) or maybe on medications that affect fluid balance (like diuretics) or cardiovascular function. We need to know if any interactions could amplify or diminish taurine’s effect. As noted, caffeine is one that has been studied recently: a combination of moderate caffeine and taurine still resulted in improved performance and thermoregulation, indicating they can coexist [11,26,27]. What about glycerol hyperhydration (which increases body water before exercise)? If someone hyperhydrates with glycerol and also takes taurine, do they get an even larger plasma volume and sweat benefit, or is it redundant? Or consider an athlete taking beta-alanine (which can raise carnosine in muscles to buffer pH) beta-alanine competes with taurine for absorption in muscle, so chronic beta-alanine use actually lowers muscle taurine content. Could this impair taurine’s effectiveness in those athletes? This intersection has not been explored. In a medical context, if a person is on a medication that affects thermoregulation (like certain antidepressants or anticholinergic drugs), would taurine help counteract those side effects? These questions could guide future specialized studies or at least warrant cautionary notes until more is known.

Laboratory studies often use fixed-intensity exercise to exhaustion, which is a controlled way to assess physiological changes but not directly representative of competitive sports or work scenarios. More research is needed on how taurine affects self-paced performance (like a time trial or a game), tactical decision-making, or technical skills under heat. For instance, does taurine help a team sport athlete maintain higher intensity in the later stages of a match in the heat? Does it reduce errors or mental lapses compared to a placebo? Field studies could be done in actual sport competitions or simulated work tasks. Moreover, monitoring actual incidence of heat illness in large cohorts using taurine would be the ultimate test of its protective value. While inducing heat stroke in a study is obviously unethical, observational data or retrospective analyses (if taurine is adopted by some groups) could be valuable.

If organizations begin to consider taurine as part of heat management, research should also address implementation aspects: What is the best way to deliver it (pills, drink mix, fortified foods)? How do we ensure compliance and proper timing? Are there any subjective side effects that could affect compliance (e.g., some people report mild stomach upset from amino acid supplements, though taurine is usually well tolerated)? Additionally, cost–benefit analysis in occupational settings might be warranted—taurine is cheap per dose, so it is likely cost-effective, but demonstrating reduced heat-related downtimes or medical events would solidify an argument for its use by employers.

To illustrate taurine’s impact in context, consider the concept of the critical environmental limit for heat stress, often expressed as the point at which heat gains exceed heat losses (the threshold of compensability) [7]. The measure P_crit (critical ambient vapour pressure) represents the ambient water vapour pressure (humidity) threshold—at a given temperature and metabolic heat production—beyond which the body can no longer maintain a steady-state core temperature (Table S2). In one study, taurine supplementation significantly increased P_crit from ~21.7 mmHg to ~25.0 mmHg, indicating that participants could tolerate a higher ambient humidity (i.e., higher vapour pressure; more challenging conditions) before crossing into uncompensable heat stress. This kind of outcome is very tangible for applied use: it quantifies how much more environmental stress (heat/humidity) one can handle with an intervention. Future research might use such metrics to compare interventions or to model how taurine might allow for longer work durations or higher work rates in various climates. Developing a conceptual model or figure that integrates taurine into the heat strain equation could be a useful tool for education and hypothesis generation. For example, one could depict how acclimation shifts the core temperature curve, how cooling shifts it, and how taurine shifts it, and then consider combined effects. This can guide where the gaps in knowledge are—e.g., does taurine simply parallel acclimation or does it interact in a non-additive way?

In conclusion, on limitations: While early evidence paints taurine as a promising, multifaceted aid for heat adaptation, significant questions remain. Addressing these through systematic research will help determine the true scope of taurine’s usefulness. The next few years may see studies that not only replicate current findings but also push into new territory: multi-day endurance events, team sports, military training exercises, and even clinical trials for prevention of heat illness. By resolving these unknowns, we can refine guidelines for taurine use (or identify situations where it should not be used). The multi-level mechanistic pathways are detailed in Table 2. Furthermore, an evidence map of existing human studies of oral taurine under environmental heat stress is provided in Table 1, and the practical interactions between taurine and established heat-mitigation strategies are detailed in Table 3 (see also Table S3).

**Table 1:** Human studies of oral taurine under environmental heat stress (evidence map; in-text).

Study (Year)	Country	Registration	Design & Blinding	*n* (♂/♀), Age (y)	Training Status	Environment (°C/%RH/WBGT)	Airflow/Clothing	Exercise Protocol & Intensity	Taurine Protocol (Dose, Timing, Form)	Comparator(s)/Co-Ingestion	Hydration Control	Outcomes & Measurement	Key Quantitative Findings
[28]	NR	NR	Randomized, double-blind, crossover; allocation concealment NR; washout NR	11 (11/0), 24 ± 3	Trained cyclists	35/40/NR	Fans NR; light kit	Cycling to exhaustion (fixed intensity)	50 mg·kg^−1^ (~3–4 g), 2 h pre; form NR	Placebo; co-ingestion NR	Standardized fluids reported	Performance: TTE; Tc: site NR; sweat: local ventilated capsule	TTE ↑ (high) ≈10%; end-exercise Tc ↓ (low) ≈0.4 °C; local sweat ↑ ≈12.7% vs. PLA
[13,28]	NR	NR	Randomized, double-blind, crossover; allocation concealment NR; washout NR	15 (12/3), age NR	Trained adults	33/30 → 70/NR	Airflow NR; cycling kit	Prolonged cycling; progressive humidity (heat-balance test); intensity NR	6 g·day^−1^ for 8 days (loading), timing NR; form NR	Placebo; co-ingestion none	Fixed intake across arms reported	Heat balance: net heat storage; sweat: whole-body washdown + local; Tc: site NR; skin BF: method NR	Evaporative heat loss ↑ ≈27%; net heat storage ↓ ≈72%; earlier & higher sweating (↑ active glands); late-exercise Tc ↓ ≈0.3 °C; skin BF ~no change
[13]	NR	NR	Randomized crossover (multi-arm); blinding NR; allocation concealment NR; washout NR	12 (12/0), age NR	Physically active	35/65/NR	Airflow NR; clothing NR	Cycling at ~70% VO_2_max to exhaustion	1.5 g, 1.5–2 h pre; form NR	Placebo; caffeine arm (dose NR); taurine + caffeine	Standardized fluids reported	Performance: TTE; Tc: site NR; Lactate: assay NR	TTE ↑ vs. PLA (TAU > CAF); Tc ↓ (large standardized effect, d ≈ 1.9); post-exercise lactate ↓

General note. Tc = core temperature; RH = relative humidity; WBGT = wet-bulb globe temperature; BF = blood flow; PLA = placebo; CAF = caffeine; NR = not reported. Symbols: ↑ = increase; ↓ = decrease.

**Table 2:** Mechanism matrix: multi-level pathways by which taurine may enhance thermoregulation (in-text).

Mechanism Domain	Level (CNS/Peripheral/Systemic)	Primary Pathway(s)	Representative Evidence	Measurement Methods	Strength/Consistency	Translational Implication
Sweating threshold reset	CNS (hypothalamus)	Taurine → GlyR/GABA_A activation → lower sudomotor set-point	Animal CNS studies; human pattern (earlier sweating in [13,28]	Animal microdialysis/infusion; human onset Tc	Moderate	Earlier sweat onset → more time in compensable zone
Eccrine gland recruitment/output	Peripheral (skin)	↑ Active gland count; ↑ local & whole-body sweat	Human trials [13,28]	Ventilated capsule; whole-body washdown	Moderate	Greater evaporative heat loss at given metabolic load
AVP/ADH modulation	CNS/systemic	Taurine may blunt AVP; ADH can suppress sweat or ↑ [Na^+^]	Animal & correlational human	Plasma/urine osmolality; AVP assays NR	Limited/mixed	Possible higher volume (more dilute) sweat; human causal confirmation needed
Osmolyte/cell-volume effects	Systemic/cellular	Taurine as compatible osmolyte; transient PV buffering	Human taurine kinetics; biochemistry	Plasma taurine/osmolarity	Limited	Do not rely on this for hydration; fluids + Na^+^ required
Mitochondrial/antioxidant support	Cellular (muscle/heart/brain)	Stabilize ΔΨm; reduce oxidative stress	Animal/in vitro	Mito function assays NR	Limited	May reduce cellular thermal/oxidative strain
Substrate utilization & Ca^2+^ handling	Muscle	↑ Fat oxidation; improved excitation–contraction coupling	Human (temperate) + mechanistic	RER; EMG/Ca^2+^ proxies NR	Moderate (non-heat)	Minor heat-production impact; endurance support
Skin blood flow	Peripheral	Vasoactive effects uncertain in heat	Human estimate [13]	LDF/plethysmography NR	Limited	Primary benefit is sweating, not skin BF

Notes. PV = plasma volume; LDF = laser Doppler flowmetry; RER = respiratory exchange ratio; NR = not reported. Symbols: ↑ = increase.

**Table 3:** Interaction matrix—taurine × acclimation, cooling, hydration, and context (in-text).

Co-Intervention/Context	Net Interaction	Physiological Rationale	Practical Integration	Risks & Mitigations	Evidence Basis
Heat acclimation	Synergy	Both ↓ sweating threshold; acclimation ↑ sweat capacity & [Na^+^] reabsorption	Use taurine early in acclimation or when time-limited; complete full acclimation block	↑ Sweat losses → plan electrolytes (Na^+^)	Human taurine trials + acclimation literature
Pre-cooling (immersion/ice slurry)	Complementary	Start cooler (more heat storage); taurine triggers earlier sweating as Tc rises	Pre-cool; ingest taurine 90–120 min pre start; monitor RPE/Tc	Avoid over-cooling that suppresses sweating at onset	Cooling meta-analyses; taurine human trials
Per-cooling (during exercise)	Context-dependent	Cold drinks can reduce sweat reflex in dry heat; valuable in humid heat	Use ice slurry mainly in humid/low-evaporation settings (breaks)	Cooling can mask thirst → track body mass	Cooling guidelines; taurine thermoreg rationale
Cooling garments/neck coolers	Additive (comfort)	External heat removal ↓ skin T°; taurine maintains evaporation capacity	Use during warm-ups/breaks; maintain airflow	If evaporation isblocked, rely more on external cooling	Garment studies; taurine trials
Hydration & electrolytes	Essential enabler	Taurine ↑ sweat volume	Start euhydrated; increase fluids ~5–15%; include sodium	Avoid water-only strategies (hyponatremia risk)	Sports hydration consensus
Encapsulating PPE	Potentially antagonistic	Evaporation impaired → extra sweat ≠ cooling	Prioritize microclimate cooling; consider deferring taurine if no evaporation possible	Faster dehydration → frequent fluid breaks	PPE heat research
Caffeine co-ingestion	Mixed	Caffeine may ↑ Tc; taurine tends to ↓ Tc	If Tc control is priority, limit high caffeine doses with taurine	Jitters/sleep risk; careful dose-timing	Caffeine heat data; taurine trials; broader caffeine performance literature (32)

Notes: Tc = core temperature; PPE = personal protective equipment; Na^+^ = sodium. Symbols: ↑ = increase/enhancement; ↓ = decrease/reduction.

The evidence to date is encouraging, but it is the continuing research—including field trials and mechanistic studies, that will ultimately determine how widely taurine will be adopted in practice. If findings remain positive, it is conceivable that in a few years taurine could be as common as electrolyte drinks in the toolkit for heat management. In the meantime, those at the forefront of sports science and occupational health can consider taurine as an emerging option, applying it judiciously and monitoring outcomes. The overarching goal remains the same: to help humans perform optimally and safely even under the most sweltering conditions. Taurine, as part of an integrative heat adaptation strategy, could significantly contribute to this goal by fortifying the body’s resilience to heat and extending the boundaries of thermal performance [6,7].

## 8. Conclusions

Adapting to exercise and work in the heat requires an integrated strategy built on heat acclimation, appropriate cooling, and individualized hydration and electrolyte replacement. Although human evidence remains limited, taurine may serve as a practical adjunct to support heat balance and sudomotor responses—particularly when full acclimation is not feasible.

Given that taurine may increase sweating, any implementation should be paired with proactive fluid and sodium planning in line with established hydration best practices. Practical guidance for athletes and workers is summarized in Table 4. Adequately powered field studies are now required to confirm efficacy, establish precise dose–response and timing, and clarify safety across diverse populations and real-world contexts.

## Figures and Tables

**Table 4:** Implementation and safety guide for athletes and heat-exposed workers (in-text).

Use Case	Dose & Timing	Likely Responders	Monitoring & Adjustments	Contraindications/Cautions	Regulatory/Safety
Acute event day	3–6 g (≈40–50 mg·kg^−1^) 90–120 min pre	Non-/partially acclimated; hot & humid events; limited airflow	Pre → post body mass change; Tc/RPE if available; increase fluids ~5–15%; add Na^+^; include sodium plan (sweat salt loss may rise with higher sweat volume)	Kidney disease/meds → medical advice; split dose if GI upset; caution in prolonged exercise or prior heat illness	Not prohibited; generally safe ≤ ~3 g·day^−1^ chronically; short-term use up to 6 g
Short-term loading (camp/heat block)	3 g·day^−1^ for 5–8 days	Repeated hot sessions (camps/tournaments)	Plan electrolytes; cooling in breaks; thirst education	Avoid when evaporation is impossible (fully encapsulated PPE) unless robust cooling	NR
Occupational (military, firefighting, construction)	3–4 g at shift start; consider split dosing on long shifts	Roles with intermittent ventilation	Ensure water + electrolytes accessible; supervisor monitoring; emphasize sodium replacement and supervisor-enforced breaks	Not a substitute for work–rest/cooling policies; do not use to “push through” heat strain	NR
Co-ingestion policy	Limit high-dose caffeine when Tc control is priority	NR	NR	NR	NR
Intermittent/team sport (breaks available)	3–6 g (≈40–50 mg·kg^−1^) 90–120 min pre; consider 1–2 g top-up only if protocol is studied	Hot & humid matches; limited acclimation time; high-sweating responders	Break-time cooling + planned drinking; monitor body mass change; avoid relying on “feels cooler”	Caution if heat illness history; avoid masking symptoms (lower RPE) without objective checks	NR
Protective clothing/low evaporation (PPE, encapsulation)	Avoid routine use as a stand-alone strategy when evaporation is severely constrained	High-heat occupations with impermeable PPE	Prioritize work–rest cycles, active cooling, and medical monitoring per policy	Higher risk of dehydration/sodium loss without proportional cooling benefit	NR

Notes. GI = gastrointestinal; RPE = rating of perceived exertion; NR = not reported.

## Data Availability

No new data were created or analyzed in this narrative review. All information synthesized is derived from published sources and is included in the article and Supplementary Materials. Further inquiries can be directed to the corresponding author.

## Supplementary Materials

The following supporting information can be downloaded at https://www.mdpi.com/article/10.3390/nu18040592/s1, Table S1: Heat-balance framework and the critical environmental limit (Pcrit)/threshold of compensability; Table S2: Multi-level mechanistic pathways by which taurine may modulate heat tolerance; Table S3: Interaction matrix: taurine × established heat-mitigation strategies; Table S4: Fluid and electrolyte adjustment framework when taurine increases sweating; Figure S1: PRISMA-compliant literature identification and selection process (schematic).
