# Supplementary material for: Taurine Supplementation and Human Heat Tolerance: Mechanisms, Evidence, and Integration with Heat Acclimation, Cooling, and Hydration
Source: Nutrients. 2026 Feb 11;18(4):592. doi: 10.3390/nu18040592 (PMC12943169; doi:10.3390/nu18040592)
Supplement: Supplementary file 1 [file nutrients-18-00592-s001.zip › nutrients-4103925-Supplementary_Materials_Taurine_Heat_Tolerance.pdf]

Supplementary Materials

for the narrative review: "Taurine Supplementation and Human Heat Tolerance: Mechanisms, Evidence, and Integration with Heat Acclimation, Cooling, and Hydration."

Section I. Methodological Framework

Figure S1. PRISMA-compliant literature identification and selection process (schematic)

A targeted evidence identification strategy was used to capture seminal human trials and relevant mechanistic models for taurine and heat tolerance. The PRISMA-style workflow comprised:

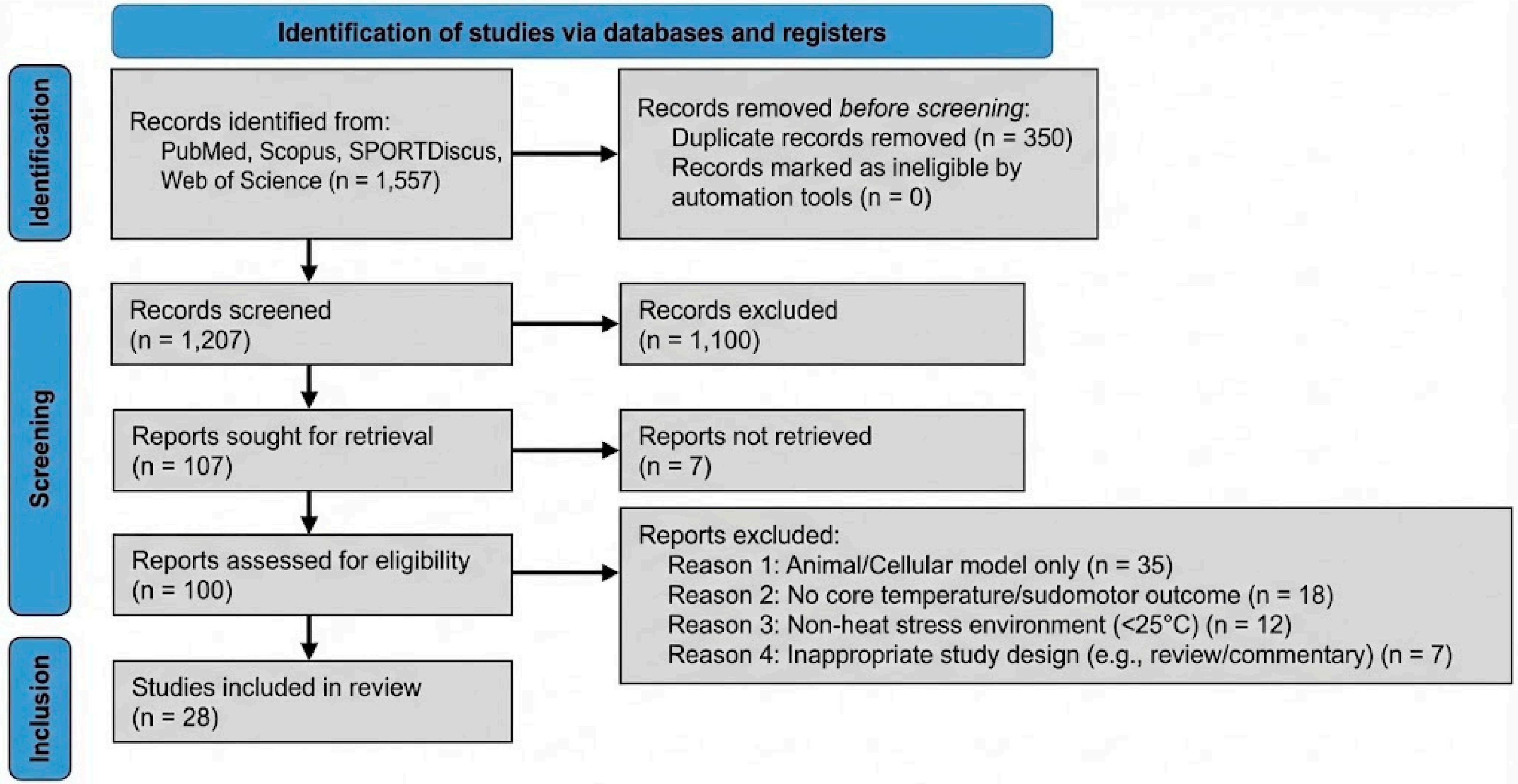

Figure S1. PRISMA 2020 Flow Diagram for Taurine and Heat Adaptation Evidence Synthesis

Table S1. Heat-balance framework and the critical environmental limit (Pcrit) / threshold of compensability

Taurine's thermoregulatory value is interpreted through **compensability**: thermal equilibrium can be maintained when the evaporative requirement (**Ereq**) does not exceed maximum evaporative capacity (**Emax**). When **Ereq = Emax**, conditions reach a critical vapor pressure (**Pcrit**), beyond which net heat storage (**S**) rises and **Tcore** increases.

Parameter	Definition	Canonical form	Interpretation for taurine integration
Heat storage (S)	Rate of body heat storage; positive values indicate rising body heat content.	$S = M - W \pm (R + C) - Esk - Eres$	A taurine-driven increase in sweating can increase Esk (evaporative heat loss), reducing S and slowing Tcore rise.
Metabolic heat production (M)	Heat produced by metabolism during exercise.	$M \approx VO2 \times$ energy equivalent	Taurine is not expected to directly reduce M at a fixed workload; benefits occur mainly by increasing heat dissipation and/or lowering perceived strain.
External work (W)	Mechanical work performed; subtracts from metabolic energy that becomes heat.	W = power output	Performance changes (pacing) alter M and W simultaneously; reporting power/pace is essential to interpret thermoregulatory responses.
Dry heat exchange (R + C)	Radiative and convective heat transfer.	$R + C = h\,(Tskin - Ta)$	Taurine does not directly change R + C; however, changes in skin blood flow and Tskin can shift gradients.
Evaporative heat loss (Esk)	Heat loss via sweat evaporation.	$Esk = he \times \omega \times (Psk - Pa)$	Taurine may increase ω (skin wettedness) via sweat output; benefit is greatest when evaporation is possible (not fully humid/encapsulated).
Respiratory heat loss (Eres)	Heat exchange via respiration.	$Eres = f(VE, Ta, RH)$	Typically smaller than Esk during exercise; report ventilation context if mechanistic interpretation is required.
Evaporative requirement (Ereq)	Evaporation needed to maintain thermal steady state.	$Ereq = Mnet - (R + C)$	Higher workloads increase Ereq; taurine's value increases when Esk can be elevated without excessive dehydration.
Maximum evaporative capacity (Emax)	Max evaporation allowed by environment and clothing.	$Emax = he \times (Psk - Pa)$	In humid/uncompensable heat, Emax is constrained; more sweating may raise fluid loss without proportional cooling.

Critical vapor pressure (Pcrit)	Condition where Ereq equals Emax (threshold of compensability).	Ereq = Emax	If taurine increases Esk effectively, it can shift the operating point away from Pcrit, delaying uncompensable strain—context dependent.

Operational notes for practitioners (units and reporting)

- Report environment as **Ta (°C)**, **RH (%)**, and if possible **WBGT** and **air velocity ($m \cdot s^{-1}$)**.
- Report **Tcore site** (rectal/esophageal/intestinal) and sampling interval (sites differ in response kinetics).
- Report sweating as **whole-body** (mass change corrected for intake/urine) or **local** (capsule), with units ($L \cdot h^{-1}$ or $mg \cdot cm^{-2} \cdot min^{-1}$).
- Note **clothing/PPE** (evaporative resistance) because it materially alters Emax.

Section III. Mechanistic Synthesis Matrix

Table S2. Multi-level mechanistic pathways by which taurine may modulate heat tolerance

This table summarizes candidate mechanisms and indicates likely direction of effect, markers, and translation relevance.

Mechanistic domain	Putative taurine action	Expected direction	Candidate measures/markers	Translation relevance
Central thermoregulation	Neuromodulatory effects (e.g., inhibitory tone) influencing thermal perception/drive.	↓ thermal discomfort / altered autonomic balance	Thermal sensation, RPE, autonomic indices	May support pacing tolerance and subjective heat strain management.
Sudomotor control	Earlier sweat onset / altered sweat sensitivity to rising Tcore.	↑ sweat rate and/or earlier onset	Sweat onset threshold, local/whole-body sweat rate	Useful in compensable heat where evaporation is available.

Osmoregulation / cell-volume	Taurine as a compatible osmolyte supporting cellular hydration and volume regulation.	↑ cellular resilience to osmotic stress	Plasma osmolality, hematocrit/hemoglobin, cell-volume markers	May preserve function under dehydration/heat stress, but requires adequate fluid strategy.
Cardiovascular stability	Potential attenuation of cardiovascular drift via fluid balance and strain perception.	↓ HR drift (context-dependent)	HR, stroke volume surrogates, plasma volume estimates	Helps interpret whether performance changes are "strain-limited."
Mitochondrial / oxidative stress	Antioxidant/mitochondrial buffering effects (mainly mechanistic evidence).	↓ oxidative perturbation	Oxidative stress panels, mitochondrial markers	Supports plausibility; direct translation to endurance heat performance needs human confirmation.

Note: ↓ Increase, ↓ decrease

Section IV. Interaction Matrix and Implementation Logic

Table S3. Interaction matrix: taurine × established heat-mitigation strategies

This matrix summarizes expected interactions, mechanisms, and practical integration.

Strategy	Net interaction	Mechanistic rationale	Practical integration
Heat acclimation	Synergistic / additive (plausible)	Both can lower sweat threshold and improve heat dissipation; taurine may provide additional sudomotor drive.	Consider taurine as an adjunct when acclimation time is limited; verify via sweat-rate monitoring.
Pre-cooling	Complementary	Pre-cooling reduces thermal strain early but can blunt sweating; taurine may offset delayed sudomotor activation as Tcore rises.	Time taurine ingestion sufficiently pre-start (e.g., 60–120 min) if used; monitor hydration needs.
Per-cooling	Context-specific	Cold fluid/ice can reduce strain but may suppress sweating in dry heat; in	Prioritize per-cooling in humid/uncompensable heat; taurine

		humid heat, cooling can be beneficial when evaporation is limited.	use should be paired with a hydration plan.
Hydration/electrolytes	Conditional dependency	If taurine increases sweating, dehydration risk rises without fluid/electrolyte replacement.	Adjust fluid and sodium to measured sweat rate; avoid "one-size" dosing.
PPE/encapsulation	Potentially antagonistic	If evaporation is blocked, more sweat does not equal more cooling; raises fluid loss without benefit.	Use cautiously (or defer) in heavy/impermeable PPE contexts unless cooling is engineered.

Section V. Quantitative Applied Guidelines

Table S4. Fluid and electrolyte adjustment framework when taurine increases sweating

When taurine meaningfully increases sweat output, rehydration plans should be scaled to match measured sweat rate and sodium losses. Values below are starting points; individualization via sweat testing is preferred.

Context	Predicted sweat impact	Adjusted rehydration goal	Sodium recommendation
Acute endurance (hot–dry)	Local sweat rate may rise if evaporation remains effective	Increase fluids progressively; aim to keep body mass loss generally <2%	Typical range ~300–600 $mg \cdot h^{-1}$ (adjust to sweat sodium if known)
Prolonged exposure (hot–humid)	Whole-body loss may rise but cooling benefit is constrained	Match intake to sweat rate as tolerated; avoid over-drinking	Consider higher sodium concentration when losses are high to reduce hyponatremia risk
Team/intermittent sport	Variable sweat loss across bouts	Add fluids at breaks; prioritize palatable electrolyte fluids	Monitor cramps/dizziness; sodium strategy should reflect session duration and losses
